# Should Hepatitis B Serosurveys Methodology Be Reconsidered?

**DOI:** 10.4269/ajtmh.2010.09-0488

**Published:** 2010-01

Dear Sir:

With interest, we read the paper by Pereira and others^1^ and would like to take this opportunity to comment on the accuracy of hepatitis B serosurveys according to the current knowledge of the biology and natural history of the hepatitis B virus (HBV).

Although the presence of HBsAb has classically been thought to preclude the possibility of active HBV infection, studies in recent years have suggested that as many as 10–25% of patients with positive HBsAg also have positive HBsAb.^2^ Therefore, a methodological approach that assumes that the presence of HBsAb excludes chronic active HBV infection may be inaccurate.

Another important issue is the necessity to describe the prevalence and natural history of occult hepatitis B, which is defined as serologically undetectable HBsAg despite the presence of HBV DNA in the serum or liver. This condition has seldom been studied in the general population. One study in an HBsAg-negative Canadian Inuit community found a prevalence of 18% in individuals with anti-HBc antibodies and 8% in HBV-seronegative individuals,^3^ whereas another study found it in 16% of HBV/hepatitis C virus (HCV)-negative, healthy Korean subjects with normal transaminase values.^4^

We also have to consider the potential clinical relevance of this situation in the future, because these patients may develop HBV reactivation on chemotherapy or immune suppression, transmit HBV through blood transfusions or organ transplants, or even have increased risk of hepatocelular carcinoma.^5^ Although laborious and economically challenging, especially in developing countries, it is of palmary importance to address the coexistence of HBsAg and HBsAb and the prevalence of occult infection to have a better idea of the real prevalence and relevance of the hepatitis B virus in areas thought to be of low or moderate endemicity.

Jorge Luis Salinas

University of Alabama at Birmingham

1530 Third Avenue, South, BDB 327

Birmingham, AL 35294-0012

E-mail: jsalinas@uab.edu

Martin Rodriguez

University of Alabama at Birmingham

1900 University Boulevard, THT 229

Birmingham, AL 35294-0006

E-mail: mrodri2@uab.edu
